# Complex Particulate Biomaterials as Immunostimulant-Delivery Platforms

**DOI:** 10.1371/journal.pone.0164073

**Published:** 2016-10-07

**Authors:** Débora Torrealba, Joaquin Seras-Franzoso, Uwe Mamat, Kathleen Wilke, Antonio Villaverde, Nerea Roher, Elena Garcia-Fruitós

**Affiliations:** 1 Institut de Biotecnologia i de Biomedicina, Universitat Autònoma de Barcelona, Cerdanyola del Vallès, Spain; 2 Departament de Genètica i de Microbiologia, Universitat Autònoma de Barcelona, Cerdanyola del Vallès, Spain; 3 CIBER de Bioingeniería, Biomateriales y Nanomedicina (CIBER-BBN), Cerdanyola del Vallès, Spain; 4 Departament de Biologia Cel·lular, Fisiologia Animal i Immunologia, Universitat Autònoma de Barcelona, Cerdanyola del Vallès, Spain; 5 Division of Structural Biochemistry, Priority Area Asthma & Allergy, Research Center Borstel, Airway Research Center North (ARCN), Member of the German Center for Lung Research (DZL), Borstel, Germany; Institute of Infectiology, GERMANY

## Abstract

The control of infectious diseases is a major current challenge in intensive aquaculture. Most commercial vaccines are based on live attenuated or inactivated pathogens that are usually combined with adjuvants, oil emulsions being as the most widely used for vaccination in aquaculture. Although effective, the use of these oil emulsions is plagued with important side effects. Thus, the development of alternative safer and cost-effective immunostimulants and adjuvants is highly desirable. Here we have explored the capacity of inclusion bodies produced in bacteria to immunostimulate and protect fish against bacterial infections. Bacterial inclusion bodies are highly stable, non-toxic protein-based biomaterials produced through fully scalable and low-cost bio-production processes. The present study shows that the composition and structured organization of inclusion body components (protein, lipopolysaccharide, peptidoglycan, DNA and RNA) make these protein biomaterials excellent immunomodulators able to generically protect fish against otherwise lethal bacterial challenges. The results obtained in this work provide evidence that their inherent nature makes bacterial inclusion bodies exceptionally attractive as immunostimulants and this opens the door to the future exploration of this biomaterial as an alternative adjuvant for vaccination purposes in veterinary.

## Introduction

The industrialization of fish production has raised numerous issues related to intensive exploitation. One of the most important challenges is the control of infectious diseases, which are easily transmitted under high-density rearing conditions, leading to significant economic losses [1, 2]. Currently available commercial vaccines are made of live attenuated or inactivated pathogens [1, 3]. However, vaccines based on live attenuated bacteria are not widely used for safety reasons, while the existing vaccines based on inactivated pathogens are generally weakly immunogenic. This limitation associated with inactivated vaccines is frequently overcome by the co-administration of immunostimulants or adjuvants [4] that are most widely applied as oil emulsions [5]. Even so, the use of oil emulsions to get a successful prophylaxis has important side effects. In this context, many other potential immunostimulants are being studied, including immune stimulating complexes (ISCOMs), beta-glucans, poly (I:C), lipopeptidases, lipopolysaccharide (LPS), flagellin, CpG and cytokines. Although some of these show promising features, in general terms, the obtained results vary considerably. For example, the protective effect of LPS against fish diseases has been frequently proven *in vivo* after challenge with certain bacterial pathogens [6–8], but not with others [9, 10]. This could be partially explained by the origin and purity of the LPS used [11]. Additionally, Mackenzie *et al*. (2010) concluded that variations in the amount of contaminants such as peptidoglycan (PGN) and nucleic acids clearly determine the extent of stimulation by the different LPS samples tested. Interestingly, they point out that an enhancement of the immune response after using LPS as an immunomodulator is the result of the effect of the complex mixture in crude LPS [9]. Moreover, it has been proven that non-protective doses of LPS, when combined with poly (I:C), exert a clear protection against *Pseudomonas aeruginosa* when encapsulated in nanoliposomes [12]. Thus, considering that a combination of immunostimulants has a powerful effect and that it would be highly convenient to develop a general immunostimulant able to release the active molecule slowly at lower doses, bacterial inclusion bodies (IBs) appear to be a promising alternative.

IBs are mechanically stable, non-toxic protein-based particulate biomaterials produced in recombinant *Escherichia coli* cells under overexpressing conditions [13]. This biomaterial is mainly composed of recombinant heterologous protein, but it also contains different bacterial components such as membrane remnants, membrane bound proteins, nucleic acids, cell wall fragments and, consequently, LPS and PGN [14–16]. Noteworthy, these elements have been proven to act as strong stimulants of the fish immune system [10]. In addition, IBs show other important characteristics that make them an appealing potential immunostimulant. On the one hand, they are produced through fully scalable, simple and cost-effective biofabrication processes. On the other hand, they have been shown to be highly stable over time under different conditions and, importantly, many physicochemical parameters of the material are fully adjustable by both genetic and process engineering [17]. Thus, all these aspects make this biomaterial a highly versatile option with excellent potential to be used as a generic immunostimulant to improve vaccination effectiveness in fish against a wide range of infectious diseases.

In this study, we have explored and characterized IBs as a new immunostimulant for fish. Data presented here indicate that bacterial IBs formed by non-biologically active heterologous proteins (VP1GFP and iRFP) are excellent modulators of host defences able to protect fish against bacterial infections.

## Materials and Methods

### Bacterial strains and plasmids

The bacterial strains used in this work to produce IBs included the *E*. *coli* K-12 derivative JGT4 (ClpA^-^; *clpA*::*kan*, araD139 (*argF*-*lac*) U169 *rpsL150* relA1 *flbB5301 deoC1 ptsF25* Rbs^R^, Sm^R^) [18], referred to as ClpA^-^, and the endotoxin-free *E*. *coli* K-12 strain KPM335 (*msbA52*, *ΔgutQ*, *ΔkdsD*, *ΔlpxL*, *ΔlpxM*, *ΔpagP*, *ΔlpxP*, *ΔeptA*, *frr181*) [19]. These strains were transformed with the pTVP1GFP (Ap^R^) plasmid (GenBank acc. number KM242650) for the production of VP1GFP IBs. VP1GFP is a modular protein consisting of the green fluorescent protein (GFP) fused to the VP1 protein of the capsid of the foot-and-mouth disease virus. In addition, *E*. *coli* BL21 (DE3) cells (F^−^*ompT hsdS*_B_(r_B_^−^m_B_^–^) *gal dcm lon* λ(DE3 [*lacI lac*UV5-T7 gene 1 *ind1 sam7 nin5*]) (Novagen) were transformed with pET22b-iRFP-H6 (Ap^R^) for the production of iRFP-H6 IBs. iRFP-H6 is an infrared fluorescent protein fused to a His-tag.

### Production and purification of IBs

*E*. *coli* strains carrying the protein expression plasmids were cultured in LB medium supplemented with ampicillin (100 μg/ml). Bacterial cultures were started at an optical density at 550 nm (OD_550_) of 0.05 and incubated aerobically (250 rpm) at 37°C until they reached an OD_550_ of 0.5. Then, 1 mM isopropyl-d-thiogalactoside (IPTG) was added and protein expression was induced for 3 h. For purification of IBs, the bacterial cultures were processed through a combination of enzymatic and mechanical disruption steps. First, lysozyme (1 μg/ml) and phenylmethanesulfonyl fluoride (PMSF, 0.4 mM) were added to cell cultures and incubated at 37°C for 2 h and 250 rpm. Then, the cells were frozen and thawed, followed by addition of Triton X-100 (0.2% (v/v)) and incubation at room temperature (RT) for 1 h with gentle agitation. IBs were harvested by centrifugation and resuspended in PBS using one tenth of the original culture volume. Next, samples were treated with 0.6 μg/ml DNase at 37°C for 1 h under agitation. Freeze/thaw cycles were repeated until no viable bacteria were detected. For this, 100 μl of the culture was seeded in LB plates without antibiotic and cultivated overnight at 37°C. Samples were centrifuged at 15,000 x *g* for 15 min, and pellets containing purified IBs were stored at -80°C until use. Finally, bacteria viability was assessed incubating purified IBs in DMEM with 10% of FBS without antibiotic during 5 days at 37°C. IBs were semiquantified by Western Blot densitometry (ImageJ) using anti-GFP and anti-RFP antibodies (Santa Cruz), and protein concentrations were inferred from standard curves made with GFP and iRFP soluble recombinant proteins that have been deeply characterized in our group.

### Structural characterization of IBs

VP1GFP IBs produced in ClpA^-^ (VP1GFP (ClpA^-^)), VP1GFP IBs produced in KPM335 (VP1GFP (KPM335)) and iRFP-H6 IBs produced in BL21(DE3) (iRFP-H6 (BL21(DE3)) were resuspended in deionized water at a final concentration of 1 μg/ml (quantification done by Western blot). Twenty microliters of these suspensions were incubated on silicon chips at RT for 2 min. After incubation, excess water was removed and the samples were air-dried overnight prior to Field Emission Scanning Electron Microscopy (FESEM, Zeiss Merlin) observation. FESEM images were processed using the image analysis software ImageJ. Diameter measurements of at least 105 particles per sample were carried out, and size distribution graphs were generated using Past3 software.

### Cell cultures

The zebrafish liver ZFL cells (CRL-2643, ATCC) were cultured at 28°C in a humidified atmosphere with 5% CO_2_ in Dulbecco´s modified Eagle´s medium (DMEM) containing 4.5 g/l glucose, 0.01 mg/ml insulin, 50 ng/ml epidermal growth factor (EGF), 5% (v/v) of antibiotic/antimycotic, 10% (v/v) heat inactivated fetal bovine serum (FBS) and 0.5% (v/v) heat-inactivated rainbow trout serum (TS) [12]. The rainbow trout macrophages (RT-HKM) were obtained from head kidney as described previously [20] and cultured in DMEM with 4.5 g/l glucose, 10% (v/v) heat inactivated FBS and 50 μg/ml Primocin (Invivogen) at 16°C and 5% CO_2_. Fully differentiated macrophages (day 5) were used for IB uptake and gene expression experiments.

### VP1GFP (ClpA^-^) IB uptake by zebrafish liver cells (ZFL) and rainbow trout macrophages (RT-HKM)

The uptake of VP1GFP (ClpA^-^) IBs by both zebrafish liver cells (ZFL) and rainbow trout macrophages (RT-HKM) was analyzed by flow cytometry and confocal microscopy. For the dose-response uptake assays, the ZFL and RT-HKM cells were seeded in 24-well plates and incubated with protein particles (IBs) at different doses (2, 4, 10 and 40 μg/ml) of VP1GFP (ClpA^-^) for 24 h (*n* = 4). For the time-course uptake assays, cells were treated with 20 μg/ml of VP1GFP (ClpA^-^) IBs and samples were taken at indicated times (ZFL: 2, 4, 8, 16 and 24 h post-treatment; RT-HKM: 0.5, 1, 2, 4, 8 and 24 h post-treatment; *n* = 4). After incubation, the medium was discarded, and the cells were rinsed with PBS to remove FBS. Each sample was treated with 1 mg/ml trypsin (Gibco) for 15 min to detach cells and remove externally attached IBs [21] and then centrifuged at 300 x *g* for 5 min, pellets were resuspended in 200 μl of PBS. The IBs uptake was analyzed using a FACS-Canto instrument (Becton Dickinson, USA) with a 15 mW air-cooled argon ion laser at 488 nm (GFP fluorescence excitation) and a D detector (530/30 nm band pass filter) for the detection of fluorescence emission. Both dose and time-course experiments were repeated four times, collecting 10,000 events for each sample. The results were analyzed with one-way ANOVA and a Tukey`s post-test. For confocal microscopy, the ZFL and RT-HKM cells were seeded in individual plates at 60% confluency and incubated with 20 μg/ml VP1GFP (ClpA^-^) for 24 h at 28 C for ZFL and 16°C for HKM. After three PBS washes, the nuclei and membranes were labeled with Hoechst and CellMask, respectively. The samples were examined with a Zeiss LSM 700 microscope (Germany), and image analysis was performed with Imaris (Bitplane AG, Switzerland).

### Cell toxicity assay

Sub confluent ZFL cells cultured in 96 well plates were incubated with 0.1, 1 and 10 μg per well of bacterial VP1GFP (ClpA^-^) IBs for 24 h at regular cell culture conditions, as described before in Cell cultures methodology. After incubation cell viability was assessed through MTT assay using the EZ4U kit (Biomedica, GmbH, Austria) following manufacturer’s specifications.

### Gene expression analysis

The effects of iRFP-H6 (BL21(DE3)) and LPS (LPS from *E*. *coli* 0111:B4, purified by phenol extraction. REF: L2630, SIGMA) on RT-HKM gene expression were analyzed by quantitative real-time PCR (qPCR). It is important to note that we decided to use trout macrophage primary cultures because the small size of the zebrafish did not allow obtaining enough cells for the qPCR analysis. The RT-HKM cells were seeded in 6-well plates at 80% confluency. Cells were incubated with 10 μg/ml of iRFP-H6 (BL21(DE3)) IBs or 10 μg/ml LPS for 12 h at 16°C (*n* = 3). Total RNA was extracted using TriReagent following the manufacturer’s instructions (Sigma). The concentration and quality of the RNA samples were evaluated using a Nanodrop ND-1000 spectrophotometer (Thermo Scientific, USA) and the Bioanalyser-2100 with the RNA 6000 Nano Kit (Agilent Technologies), respectively. The cDNA synthesis was performed with 1 μg of total RNA using SuperScript III reverse transcriptase (Invitrogen) and oligo-dT15 primer (Promega). The qPCR was carried out using SYBR Green I PCR Supermix (Bio-Rad), 250 nM of primers (Table 1) and 2.5 μl of diluted cDNA (1:50 for target mRNA and 1:500 for reference gene) in a final volume of 10 μl. The primers used in the present study are listed in Table 1. The gene for elongation factor 1 alpha (EF1) was used as a reference, and quantification was done according to the Livak method [22]. The experiment was repeated three times, and all samples were analyzed in triplicate by qPCR. The results were analyzed using One-way ANOVA followed by Tukey’s post test.

**Table 1:** Rainbow trout specific primers for qPCR.

Gene	Primer name	Sequence	Accession n°
Elongation factor 1 ɑ	om_EF-1	For_CAAGGATATCCGTCGTGGCA	NM_001124339.1
		Rev_ACAGCGAAACGACCAAGAGG	
Tumor necrosis factor ɑ	om_TNF-α	For_CGCTGACACAGTGCAGTGGA	NM_001124357.1
		Rev_TCCCCGATGGAGTCCGAATA	
Interleukin 1 β	om_IL-1β	For_GGAGGCAGCAGCTACCACAAA	NM_001124347.2
		Rev_CCGATTTGGAGCAGGACAGG	
Interleukin 8	om_IL-8	For_AGAATGTCAGCCAGCCTTGT	AJ279069
		Rev_TCTCAGACTCATCCCCTCAGT	
Cyclooxygenase 2	om_COX-2	For_TACCAAGCAGATCGCTGGAC	NM_001124348.1
		Rev_GCGTATGGCTTCATGGAGAA	
Matrix metalloproteinase 9	om_MMP9	For_TTCCAATTCAAGGGCAACTC	NM_001124370.1
		Rev_TCAGCCCCCACAGTTAAGAG	
Suppressor of cytokine	om_SOCS3	For_AACAACACAAGATATCAAGCTCAAG	AM748723.1
signaling 3		Rev_GAAGGTCTTGTAACGGTGAGGCAG	

### HEK-Blue LPS detection

The LPS detection assays using tenfold serial dilutions of VP1GFP (ClpA^-^), VP1GFP (KPM335) and iRFPH6 (BL21(DE3)) IBs were performed by triplicate with HEK-Blue™ hTLR4 cells in accordance with the specifications of the supplier (InvivoGen). Stimulation of these cells induces the production of the NF-κB -and activator protein-1 (AP-1)-dependent reporter secreted embryonic alkaline phosphatase (SEAP). NF-κB-dependent SEAP activity was determined by reading the absorbance at 655 nm following incubation of the samples at 37°C for 3 h in the presence of the QUANTI-Blue™ substrate. For the positive control experiments, tenfold serial dilutions of LPS from *E*. *coli* K-12 (InvivoGen) and recombinant human TNF-α (200 ng/well) (InvivoGen) were used. To determine the basal levels of SEAP (NF-κB) activity, pyrogen-free water and Dulbecco´s phosphate-buffered saline served as negative control. The positive and negative control reactions were prepared with the same number of HEK-Blue cells per well and assayed under the same conditions as for the IBs.

HEK-Blue™ Null2 cells, the parental cell line of HEK™-Blue hTLR4 cells lacking the hTLR4/MD-2 receptor complex, were used as a control in all hTLR4/MD-2 activation assays.

### Total lipid content

The total lipid content of the IBs was analyzed following the procedure described by Izard *et al* [23]. Briefly, 1 mg of IBs was lyophilized and 200 μl of chloroform were added and let to evaporate. The samples were subsequently resuspended in 100 μl of deionized water. Two milliliters of 18 M sulphuric acid were added, followed by an incubation of the samples in a boiling water bath for 10 min. As soon as the samples were cooled down, 5 ml of phosphoric acid—vanillin (0.12 g vanillin in 100 ml of 85% acid) were added and incubated at 37°C for 15 min. The absorbance was measured at 530 nm, and samples were quantified by inferring the total lipid amount out from a triolein standard curve. All assays were performed in triplicate.

### Animals

Adult wild-type zebrafish (*Danio rerio*) were maintained in a recirculation freshwater system under a photoperiod of 12:12 h light/dark cycle at 28 ± 0.5°C and fed daily with a commercial diet. All experimental procedures were approved by the Ethical Committee of the Universitat Autònoma de Barcelona (Reference number 1968 and HR-14-13) according to the International Guiding Principles for Biomedical Research Involving Animals (EU 2010/63). The infection procedure included monitoring of humane endpoints (numerical scale from 0 to 4) that requires euthanization of the animals when they show signs of infection (ethyl 3-aminobenzoate methanesulfonate, MS222 overdose of 300 ppm). We evaluate the clinical symptoms of infection as follow 0. Normal; 1. abdominal distension; 3. Loss of scales; 4. Abdominal or gill bleeding and the behavioural symptoms as 0. Normal; 1. Erratic or horizontal swimming; 2. Swimming just below the surface; 3. Sink to the bottom of the tank; 4. No touch response. When the sum of the clinical symptoms and the behavioural symptoms is between 1–3 we supervise the fish every 3 h during the day (instead of twice a day) and when it is more than 3 we euthanize the animal (MS222 overdose) and we compute it as a death.

### *In vivo* treatment of Zebrafish with VP1GFP (ClpA^-^), VP1GFP (KPM335) and iRFP-H6 (BL21(DE3)) IBs

Zebrafish (0.53 ± 0.07 g body weight) were placed into small tanks (4 L) at 28 ± 1°C 1 day before the experiments. *Pseudomonas aeruginosa* (PAO1, sub-line MPAO1; obtained from the Seattle PAO1 transposon mutant library, University of Washington) was cultured as described previously [12]. First, we evaluated a dose-response of VP1GFP (ClpA^-^) (300, 150, 75, 50 and 25 μg/ml), then, we compared the protection between VP1GFP (ClpA^-^) and VP1GFP (KMP335) (300 μg/ml), and finally, between VP1GFP (ClpA^-^) and iRFP-H6 (BL21(DE3)) (150 μg/ml) (*n* = 15). For IBs and PAO1 injection, the fish were anaesthetized with MS-222 (166 ppm) and intraperitoneally (i.p) injected with either 20 μl of IBs or PBS as a control. At 7 days post-injection, the fishes were challenged by i.p. injection with 20 μl of *P*. *aeruginosa* PAO1 (LD_50_ = 6.5 x 10^7^ cfu/animal) and their survival was followed for 7 days. Survival curves were analyzed using the Kaplan–Meier method and the statistical differences were evaluated using the log-rank test (GraphPad, USA). Relative percentage of survival (RPS) was calculated according to RPS (%) = [(1 − mortality treated group)/mortality control] × 100.

## Results

### Purification and characterization of IBs

**Fig 1:**
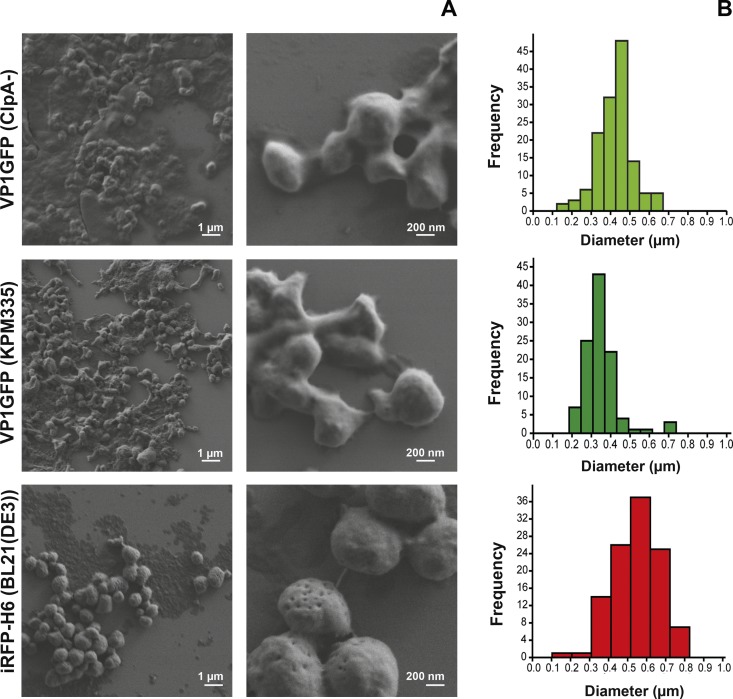
Characterization of IB particles. A) FESEM images and B) size distribution of VP1GFP (ClpA^-^), VP1GFP (KPM335) and iRFP-H6 (BL21(DE3)) IBs.

Bacterial IBs were successfully produced in *E*. *coli* and purified by a combination of mechanical and enzymatic methods. As shown in Fig 1A, the appearance of the particles differs depending on the protein and the bacterial strain used as a producer. The iRFP-H6 IBs appeared as spherical particles, whereas VP1GFP IBs exhibited, particularly in the case of those produced in KPM335, quite irregular shapes. With regard to size distributions, the IBs turned out to be polydisperse particles ranging from 100 nm to 800 nm in diameter. Nevertheless, a main peak could be clearly observed placed between 300–400 nm, 400–500 nm and 500–600 nm in diameter for the VP1GFP (KPM335), VP1GFP (ClpA^-^) and iRFP-H6 (BL21(DE3)) IBs, respectively (Fig 1A).

**Fig 2:**
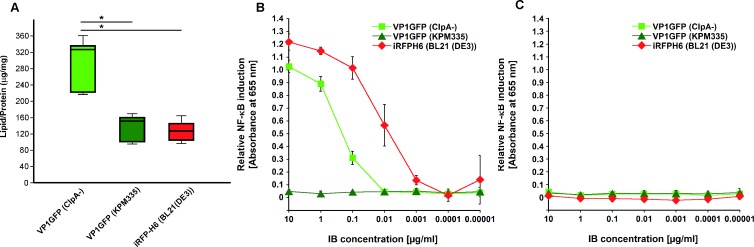
Lipid quantification and dose-response curves of NF-κB induction by IBs. A) Total lipid quantification of IB samples. Differences were analyzed using the T test, *, p<0.01. The IBs produced in *E*. *coli* strains with different LPS chemotypes were assayed with HEK™-Blue hTLR4 (B) and Null2 (C) cells for relative NF-κB induction. The absorbance values despicted in Fig 2B and 2C represent the means and standard deviations from three individual experiments. The IBs displayed negligible stimulation of the parental HEK™-Blue Null2 cell line (C), which indicates that NF-κB-dependent SEAP expression was specifically induced via the hTLR4/MD-2 signalling pathway in HEK™-Blue hTLR4 cells.

Moreover, a detailed inspection of the SEM images revealed differences in the amount of IB-associated cellular debris, which most likely is a remnant of bacterial cell membranes. Particles produced in the ClpA-defective *E*. *coli* strain showed higher amounts of cell debris co-purified with the protein particles (Fig 1A). This observation was in accordance with the values obtained for total lipid quantification of purified IBs, with VP1GFP (ClpA^-^) IBs showing the highest lipid/protein ratio (Fig 2A).

### Uptake of VP1GFP (ClpA^-^) IBs by zebrafish liver cells (ZFL) and rainbow trout macrophages (RT-HKM)

**Fig 3:**
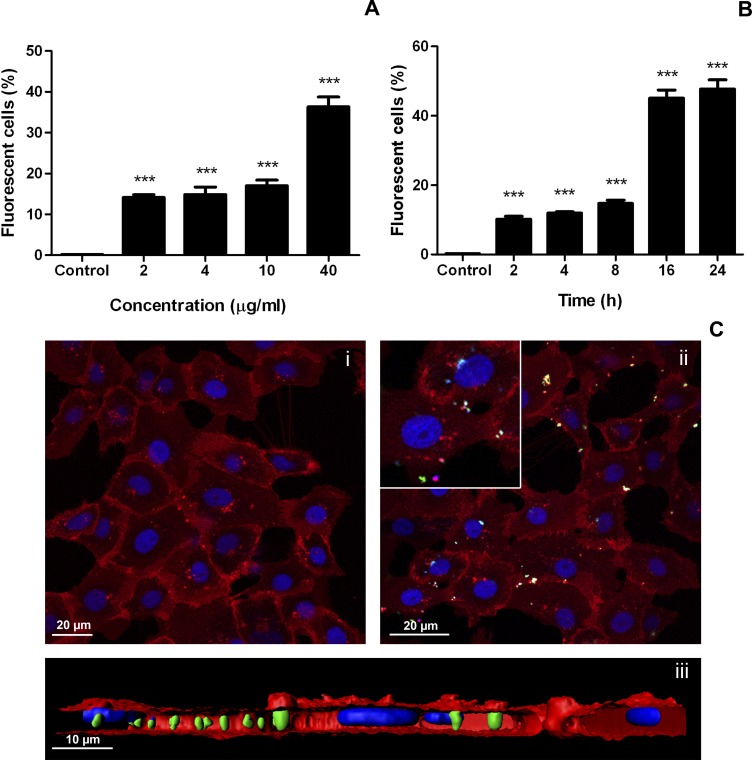
Uptake of VP1GFP (ClpA^-^) IBs by ZFL cells. A) Dose-response. Cells were incubated with 2 to 40 μg/ml of VP1GFP (ClpA^-^) IBs for 24 h. Values represent the mean and SD (*n* = 4). Differences were analyzed using One-way ANOVA and Tukey’s post test. Significant differences against control: ***, *p<*0.0001. B) Time-course. Cells were incubated with 20 μg/ml of VP1GFP (ClpA^-^) IBs for 2, 4, 8, 16 and 24 h. Values represent the mean and SD (*n* = 4). Differences were analyzed using One-way ANOVA and Tukey’s post test. Significant differences against control: ***, *p<*0.0001. C) Confocal microscopy images of VP1GFP (ClpA^-^) IB uptake by ZFL cells (green). Cells were incubated 24 h with 20 μg/ml of VP1GFP (ClpA^-^) IBs. CellMask (red) was used for plasma membrane staining and Hoechst (blue) for nuclei labeling. i. Control cells. ii. ZFL cells at 24 h post-incubation with VP1GFP (ClpA^-^) IBs. iii. 3D image reconstruction of VP1GFP (ClpA^-^) IBs uptake by ZFL cells (z-stack).

**Fig 4:**
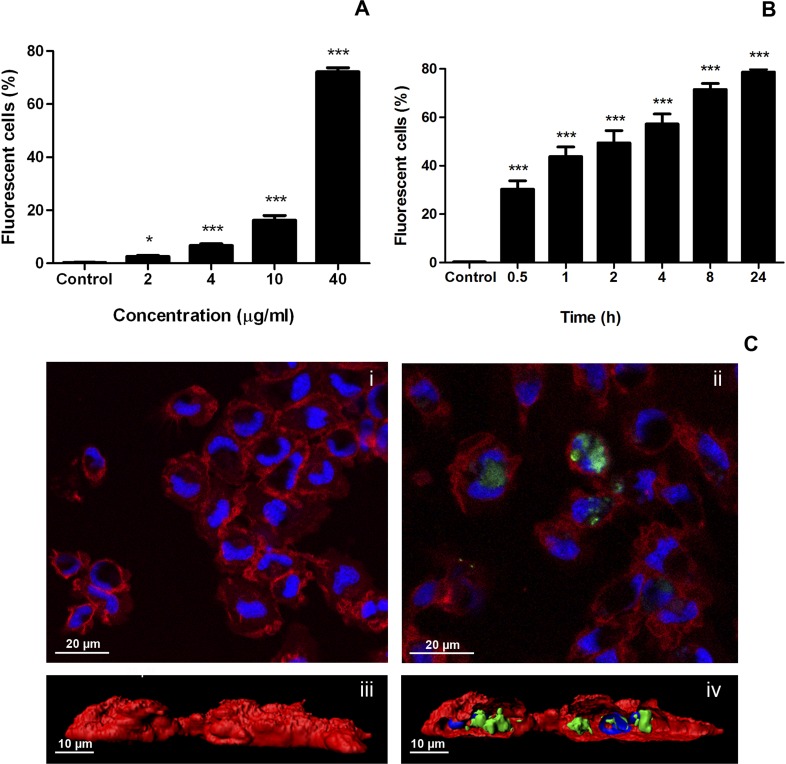
Uptake of VP1GFP (ClpA^-^) IBs by RT-HKM cells. A) Dose-response. Cells were incubated with 2 to 40 μg/ml of VP1GFP (ClpA^-^) for 24 h. Values represent the mean and SD (*n* = 4). Differences were analyzed using One-way ANOVA and Tukey’s post tests. Significant differences against control: *, *p<*0.05; ***, *p<*0.0001. B) Time-course. Cells were incubated with 20 μg/ml of VP1GFP (ClpA^-^) for 0.5, 1, 2, 4, 8 and 24 h. Values represent the mean and SD (*n* = 4). Differences were analyzed using One-way ANOVA and Tukey’s post test. Significant differences respect control: ***, *p<*0.0001. C) Confocal microscopy images of VP1GFP (ClpA^-^) IB uptake by RT-HKM cells (green). Cells were incubated 24 h with 20 μg/ml of VP1GFP (ClpA^-^). CellMask (red) was used for plasma membrane staining and Hoechst (blue) for nuclei labeling. i. Control cells. ii. RT-HKM cells at 24 h post stimulation with VP1GFP (ClpA^-^) IBs. iii. 3D image reconstruction of VP1GFP (ClpA-) uptake by RT-HKM cells. iv. 3D image reconstruction of VP1GFP (ClpA^-^) uptake by RT-HKM cells (z-stack).

One of the main properties of IBs is their ability to cross mammalian cell membranes [21, 24]. To explore the interaction of IBs with fish cells, the uptake of VP1GFP (ClpA^-^) IBs by zebrafish liver cells (ZFL) was assessed. The results showed that ZFL cells were able to uptake VP1GFP (ClpA^-^) IBs (Fig 3). The dose-response assays analyzed by flow cytometry indicated that VP1GFP (ClpA^-^) IBs tested were clearly endocyted by ZFL cells at all concentrations (14 ± 1.35% positive cells at 2 μg/ml and 35 ± 4.9% positive cells at 40 μg/ml) (Fig 3A). Additionally, IBs did not cause significant toxicity in these cells (S1 Fig), as it has been previously described for mammalian cell lines such as, HeLa, HL-60 and NIH-3T3 [24, 25]. The time-course indicated that the VP1GFP (ClpA^-^) IBs started to be endocyted by ZFL cells quite rapidly (10 ± 1.87% positive cells at 2 h post exposure), and after 24 h half of the cells contained internalized IBs (48 ± 5.9% at 24 h) (Fig 3B). To explore the interaction of IBs with professional phagocytes such as macrophages, we assessed the uptake of VP1GFP (ClpA^-^) IBs by rainbow trout macrophages (RT-HKM) (Fig 4). The results showed that trout macrophages were able to uptake VP1GFP (ClpA^-^) IBs even more efficiently than ZFL cells since, at 40 μg/ml, around 70% of trout macrophages were positive for IB uptake (72.17 ± 3.08%) (Fig 4A). A linear correlation between dose and uptake is observed in both ZFL and RT-HKM cells with a R^2^ = 0.8477 and R^2^ = 0.9993, respectively. We also observed that VP1GFP (ClpA^-^) IB started to be endocyted by RT-HKM cells faster than by ZFL cells (30.3 ± 6.99% at 0.5 h), and 24 h after exposure about 80% of the cells were positive (78.67 ± 2.23% at 24 h) (Fig 4B). Interestingly, a clear linear correlation exists between time and internalization values in the time-course experiment performed with ZFL cells (R^2^ = 0.9261) However, RT-HKM time-course fits an hyperbolic correlation (R^2^ = 0.8994). Confocal microscopy and 3D-reconstruction images enabled the visualization of VP1GFP (ClpA^-^)-agglomerates in the cytosol of ZFL and RT-HKM cells (Figs 3C and 4C). These images demonstrated the complete internalization of IBs by both cell lines (Figs 3C (iii) and 4C (ii and iv)).

### Immunization of zebrafish with VP1GFP IBs produced in *E*. *coli* strains with different LPS chemotypes

**Fig 5:**
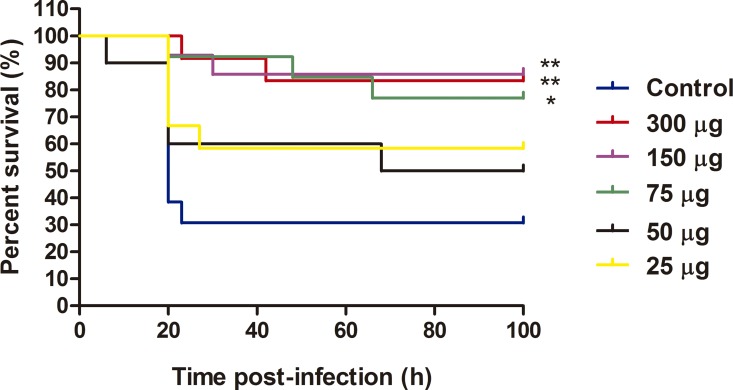
Immunization of zebrafish with VP1GFP (ClpA^-^) IBs. Survival curves of zebrafish after i.p. injection of VP1GFP (ClpA^-^) IBs at different doses (300, 150, 75, 50 and 25 μg/fish) and challenge with *P*. *aeruginosa* PAO1 (4.3x10^7^ cfu/animal) (*n* = 13). Untreated zebrafish that had been infected with PAO1 at LD_50_ were used as a mortality control. Significant differences were analyzed using the Log-rank test; **, *p<*0.01; *, *p<*0.05.

Next, to evaluate whether IBs were able to confer protection against a lethal infection *in vivo*, we used a zebrafish bacterial infection model with *P*. *aeruginosa* as an infectious agent [12]. The results showed higher survival rates when zebrafish were treated with different doses of VP1GFP (ClpA^-^) IBs, being significantly higher at 300, 150 and 75 μg (RPS of 76% at 300 μg, 79% at 150 μg and 67% at 75 μg) (Fig 5). Importantly, we did not observe any secondary effect after IB injection and animal behavior was normal after that.

**Fig 6:**
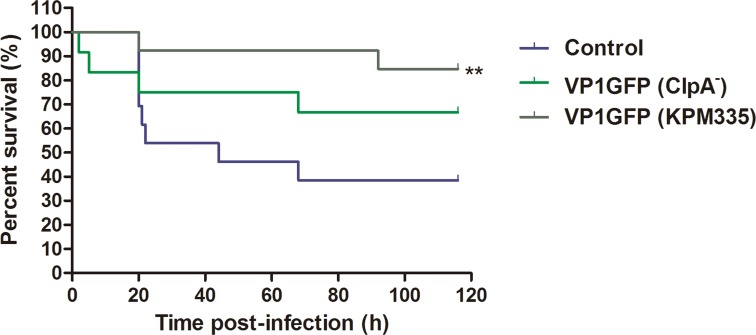
Immunization of zebrafish with VP1GFP (ClpA^-^) and VP1GFP (KPM335) IBs. Survival curves of zebrafish after i.p. injection of VP1GFP (ClpA^-^) and VP1GFP (KPM335) (300 μg/fish) and challenge with *P*. *aeruginosa* PAO1 (3.4x10^7^ cfu/animal) (*n* = 15). Untreated zebrafish that had been infected with PAO1 at LD_50_ were used as a mortality control. Significant differences were analyzed using the Log-rank test; **, *p<*0.01.

After having established the optimal dose of VP1GFP (ClpA^-^) that confer protection and since our working hypothesis was to use IBs as a general immunostimulant, we decided to analyze the specific role of LPS normally present in IBs in the observed effect. For that purpose, we compared the effects on fish survival of VP1GFP IBs produced in *E*. *coli* ClpA^-^ and *E*. *coli* KPM335. The latter strain has been demonstrated to synthesize lipid IV_A_ as the only LPS-related molecule, which lacks endotoxic activity in human LPS-responsive cells [19]. HEK-blue assay results showed that, IBs produced in KPM335 were unable to activate the hTLR4/MD-2 signaling pathway in the HEK-Blue assay (Fig 2B). In contrast, the stimulation of the HEK-Blue cell line was high when standard *E*. *coli* K-12 and B strains containing endotoxically active LPS were used (Fig 2B). Despite this difference in the endotoxic activity, the survival rate of fish immunized with IBs produced in the LPS-free strain (VP1GFP (KPM335)), achieving an RPS of 75%, was significantly higher than the control. However, no significant differences between survival rates associated with VP1GFP (ClpA^-^) IBs and VP1GFP (KPM335) IBs were observed (Fig 6).

As we have mentioned above, we also tested the total lipid content of the IBs produced in the different *E*. *coli* strains used (Fig 2A). The presence of total lipids were reduced in KPM335 (140 μg/μg lipid/protein ratio) compared to ClpA^-^ (320 μg/μg lipid/protein ratio) (Fig 2A).

### Immunization with VP1GFP (ClpA^-^) and iRFP-H6 (BL21(DE3)) IBs

**Fig 7:**
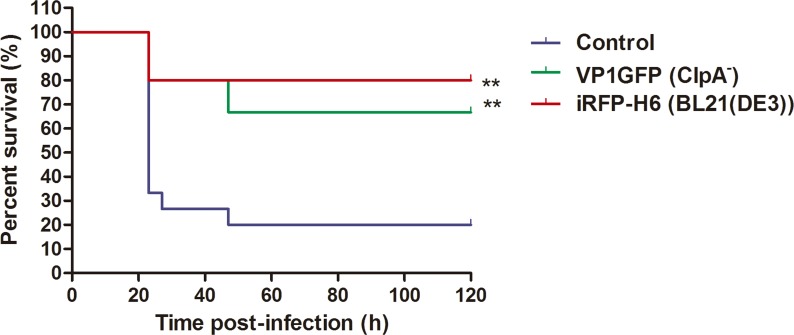
Immunization of zebrafish with VP1GFP (ClpA^-^) and iRFP-H6 (BL21(DE3)) IBs. Survival curves after i.p. injection of VP1GFP (ClpA^-^) and iRFP-H6 (BL21(DE3)) IBs at 150 μg/fish and challenge with *P*. *aeruginosa* PAO1 (4.9x10^7^ cfu/animal) (n = 15). Untreated zebrafish that had been infected with PAO1 at LD_50_ were used as a mortality control. Significant differences were analyzed using the Log-rank test, **, *p<*0.01.

In order to examine whether the amino acid sequence of the protein forming the IBs may contribute to zebrafish protection upon challenge, VP1GFP (ClpA^-^) and iRFP-H6 (BL21(DE3)) IBs were compared. Our results showed that both the VP1GFP (ClpA^-^) and the iRFP-H6 (BL21(DE3)) IBs developed comparable protection levels (RPS of 55% and 72.5%, respectively) after a lethal challenge with *P*. *aeruginosa* PAO1 (Fig 7).

### Gene expression of immune response genes in rainbow trout macrophages (RT-HKM)

**Fig 8:**
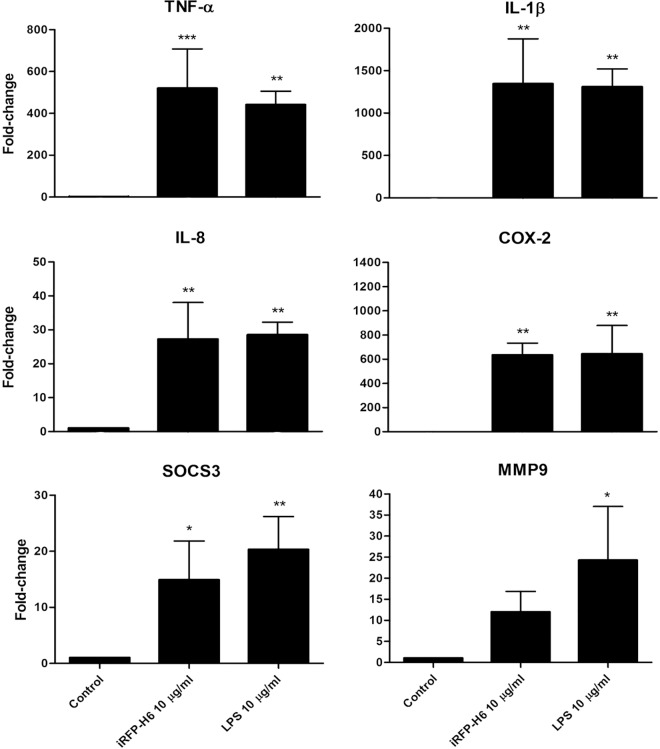
Analysis of gene expression in RT-HKM cells stimulated with iRFP-H6 (BL21(DE3)) IBs and LPS. Cells were incubated with 10 μg/ml of iRFP-H6 (BL21(DE3)) IBs and 10 μg/ml LPS for 12 h and the gene expression was analyzed by qPCR. Values represent means ± SD (*n* = 3). Significant differences against control were analyzed using One-way ANOVA followed by Tukey’s post test; *, *p<*0.05; **, *p<*0.01; ***, *p<*0.001.

To explore the ability of the IBs to stimulate the expression of immune-related genes in specific immune cells, we treated RT-HKM cells with iRFP-H6 (BL21) IBs and with LPS (dose with proven pro-inflammatory activity in trout macrophages model) as a control [20]. In general, we observed a similar increase of the gene expression in iRFP-H6 (BL21) IBs- and LPS-treated macrophages (Fig 8). The pro-inflammatory cytokines TNF-α, COX-2, IL-1β and IL-8 reached almost the same expression levels with iRFP-H6 (BL21) IBs and LPS-stimulated cells (Fig 8). As shown in Fig 8, their expression levels also increased in iRFP-H6 IBs-treated macrophages (11.9 ± 4.9 fold change for MMP9 and 14.88 ± 6.94 fold change for SOCS3), but they did not reach the same expression levels as in the LPS-treated cells (around 50% higher).

## Discussion

The development of new and effective vaccines is an increasing need for intensive aquaculture to prevent and control major diseases [2, 3]. So far, vaccines based on inactivated pathogens have been the most widely used approach to prevent important diseases. However, this strategy is inefficient in many cases, frequently requiring of high doses and/or the co-administration of adjuvant molecules to achieve the desired protection [1]. Thus, aiming to develop effective and cost-effective prophylaxis methods, future vaccine research is focused on both the improvement of vaccine potency and efficacy and the use of molecules with a general immunostimulatory profile. On the latter point, although several adjuvants have been explored, most of them show adverse side effects and/or do not show consistent levels of protection [26]. In this context, the results of this study show that bacterial IBs can be used as an alternative broad-spectrum immunostimulant-delivery platform for fish. IBs are complex, mechanically stable and functional biomaterials that combine several key properties attractive in their use as immunostimulants. Although their main component is the recombinant protein, they also contain undetermined amounts of other molecules of the bacterial production strain such as bacterial DNA, RNA and cell wall components, including LPS and PGN. Interestingly, our results prove that cell debris co-purified with IBs (all of them produced under the same growth conditions), and more specifically total lipid content, largely depends on the genetic background of the producing cells (Fig 2A). As it has been described previously, the genetic background of the producing strain, as well as the nature of the recombinant protein used to generated IBs, have also a significant impact on the final IB shape and size (Fig 1) [27, 28]. Such compositional complexity combined in a single particle with a structured form, two properties that can be easily modulated, is what makes IBs appealing as immunostimulators to enhance the immune protection. Here, we provide evidence that heterologous IBs can be used to protect zebrafish against otherwise lethal bacterial challenge (Figs 5–7). Our results suggest that none of the individual components embedded in IBs such as LPS, lipid or the recombinant protein itself, is solely responsible for induction of immune protection, but the combination of all of them in an organized and stable structured material (Figs 6 and 7). IBs are not a simple mixture of protein, LPS and other bacterial cell wall components, but protein aggregates perfectly structured forming a well-defined and resistant protein scaffold where different components are embedded, providing stability and slow release liberation to all of them [21, 29]. This novel combination of properties provides a different form of immunostimulant from a mere mix of protein, LPS and other bacterial components.

It is important to note that the use of different *E*. *coli* strains allow us to design IBs with easily modulated composition. However, we can affirm that the protective effect of the IBs in fish is independent of the LPS chemotype of the production strain (Fig 6), which means that LPS activity is not the only responsible of the protection observed. Besides, the different lipid content (Fig 2A) does not explain the slightly higher protection levels observed upon administration of VP1GFP (KPM335) IBs materials (Fig 6). Finally, the absence of significant differences between the immunoprotective capacities of these biomaterials form by two different aminoacidic sequences suggested that the nature of the protein itself does not have any effect on protection (Fig 7).

Regarding the proteins used for IB formation in this study, both model proteins are fluorescent reporters with no extra function known in fish. Nevertheless, it cannot be ruled out that, if using IBs with a specific fish protein involved in immune response (e.g. cytokines), even higher protection levels could be achieved, thus opening a new door to further tune the immunostimulant capacity of the biomaterial.

Finally, IBs have an intrinsic capacity to be efficiently endocyted in both zebrafish cells and trout macrophages. The intracellular accumulation of the structured biomaterial has already been studied in detail using a human cell line and, in general terms, the localization observed in fish cell lines (Figs 3 and 4) is perfectly consistent with the mechanism proposed in mammalian cell lines [21]. Briefly, IBs, after being intimately attached to the cell membrane, are internalized following a macropinocytic pathway [21], reaching maximum levels at 24 h (Figs 3 and 4). This could be crucial considering the localization of the TLRs involved in bacterial DNA recognition (TLR9) in endosomal compartments. The capacity of IBs to target these intracellular receptors, which recognize specific unmethylated CpG motifs prevalent in microbial but not in vertebrate genomic DNA, could lead to the activation of the TLR9 signaling pathways that ends up with the synthesis of several cytokines. In agreement with this, we observed an increase of gene expression of several markers involved in inflammation in primary cultures of trout such as TNF-ɑ, COX-2, IL-1-β, IL-8 or MMP9, all of them being crucial for the fish immune response (Fig 8). This increase is probably a combined effect of the activation of different TLRs, including those located in the endosomal membrane such as TLR9.

## Conclusions

The results obtained in this work allow us to propose IBs as a promising alternative immunostimulant to be used in intensive aquaculture. IBs are highly stable protein-based biomaterials obtained under recombinant production conditions able to provide good protection levels for zebrafish against a lethal bacterial challenge. Their inherent nature makes them exceptionally interesting, since the components embedded in a single particle allow them to increase innate fish immune protection. Moreover, IBs, in contrast to synthetic biomaterials, are natural cell components that can be easily produced by biofabrication in a cost-effective manner using recombinant bacteria as cell factories. This means that their production can be easily scaled up, becoming attractive for veterinary purposes. Besides, this study can potentially open a new field of study to take a step forward in the evaluation of this excellent biomaterial as adjuvants for vaccination purposes.

## Supporting Information

S1 Fig Toxicity in ZFL cell cultures after 24 h incubation with VP1GFP (ClpA^-^) IBs. Relative cell number of ZFL cells after the incubation with 0.1, 1 and 10 μg of VP1GFP (ClpA^-^) IBs in 96 well plates during 24 h. Control cells without IBs indicates 100% of cell viability.
(TIF)
