# Supplementary figures and images for: Complex Particulate Biomaterials as Immunostimulant-Delivery Platforms
Source: PLoS One. 2016 Oct 7;11(10):e0164073. doi: 10.1371/journal.pone.0164073 (PMC5055299; doi:10.1371/journal.pone.0164073)

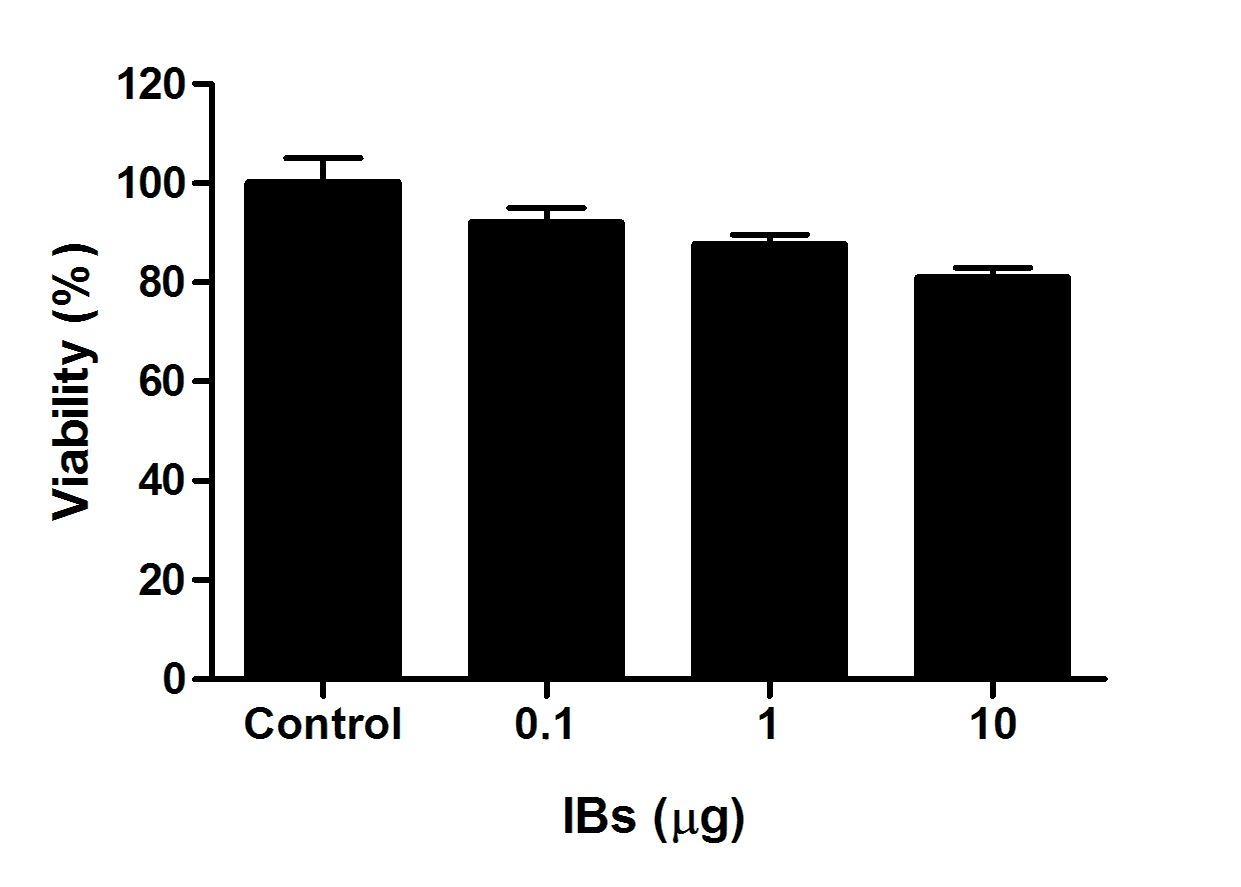

Supplement: S1 Fig — Relative cell number of ZFL cells after the incubation with 0.1, 1 and 10 μg of VP1GFP (ClpA-) IBs in 96 well plates during 24 h. Control cells without IBs indicates 100% of cell viability. (TIF) [file pone.0164073.s001.tif]
